# The Effect of Robot-Led Distraction during Needle Procedures on Pain-Related Memory Bias in Children with Chronic Diseases: A Pilot and Feasibility Study

**DOI:** 10.3390/children9111762

**Published:** 2022-11-17

**Authors:** Emma Rheel, Tine Vervoort, Anneleen Malfliet, Jutte van der Werff ten Bosch, Sara Debulpaep, Wiert Robberechts, Evelyn Maes, Kenza Mostaqim, Melanie Noel, Kelly Ickmans

**Affiliations:** 1 Pain in Motion Research Group (PAIN), Department of Physiotherapy, Human Physiology and Anatomy, Vrije Universiteit Brussel, 1090 Brussels, Belgium; 2 Department of Experimental-Clinical and Health Psychology, Ghent University, 9000 Ghent, Belgium; 3 Department of Physical Medicine and Physiotherapy, University Hospital Brussels, 1090 Brussels, Belgium; 4 Research Foundation—Flanders (FWO), 1090 Brussels, Belgium; 5 Department of Pediatrics, University Hospital Brussels, 1090 Brussels, Belgium; 6 Pediatric Department, Ghent University Hospital, 9000 Ghent, Belgium; 7 Department of Psychology, University of Calgary, Calgary, AB T3S, Canada; 8 Hotchkiss Brain Institute, University of Calgary, Calgary, AB T3S, Canada; 9 Alberta Children’s Hospital Research Institute, Calgary, AB T3S, Canada; 10 Movement & Nutrition for Health & Performance Research Group (MOVE), Department of Movement and Sport Sciences, Vrije Universiteit Brussel, 1050 Brussels, Belgium

**Keywords:** children, pain memory, humanoid robot, distraction, needle procedure, feasibility

## Abstract

The current study evaluated the feasibility and preliminary clinical impact of robot-led distraction during needle procedures in children with chronic diseases on pain-related memories. Participants were 22 children (8–12 years old) diagnosed with a chronic disease (e.g., chronic immune deficiency) and undergoing a needle procedure as part of their routine treatment. Children were randomized to the experimental group (i.e., robot-led distraction) or control group (i.e., usual care). For feasibility, we evaluated study- and needle-procedure-related characteristics, intervention fidelity and acceptability, and nurse perceptions of the intervention. Primary clinical outcomes included children’s memory bias for pain intensity and pain-related fear (1 week later). Results indicated that intervention components were >90% successful. Overall, the robot-led distraction intervention was perceived highly acceptable by the children, while nurse perceptions were mixed, indicating several challenges regarding the intervention. Preliminary between-group analyses indicated a medium effect size on memory bias for pain intensity (Hedges’ g = 0.70), but only a very small effect size on memory bias for pain-related fear (Hedges’ g = 0.09), in favor of the robot-led distraction intervention. To summarize, while feasible, certain challenges remain to clinically implement robot-led distraction during needle procedures. Further development of the intervention while accounting for individual child preferences is recommended.

## 1. Introduction

Children with chronic diseases undergo numerous needle procedures as part of their treatment [1,2,3]. Even though these procedures are often perceived as being the most distressing part of treatment, they are an essential element in the assessment and management of acute and chronic illness in children [1]. Despite the availability of evidence-based strategies for mitigating pain during medical procedures (e.g., balloon inflation [4,5], hypnosis [6,7], and topical anesthetics [8,9]), procedure-related pain, fear, and distress in children are often poorly managed, causing child suffering and caregiver distress, as well as longstanding fear and avoidance of medical procedures [1,10].

Children’s memories of painful experiences are considered critical in developing or maintaining pain problems later in life [11,12,13,14,15]. Memory representations of painful experiences are complex, including somatosensory (e.g., pain intensity), affective (e.g., anxiety), and contextual (e.g., details of the operating room) aspects of the event [16]. Such memories can be accurate when recalled levels of pain intensity or pain-related fear (e.g., one week later) are equal to reports given at the time of the painful event [17]. Yet, memories are constructed and reconstructed, and susceptible to distortion over time [18], allowing them to become biased. A negative memory bias (i.e., reporting higher pain intensity or pain-related fear during subsequent recall as compared to the initial pain report) has important implications for children’s coping during subsequent painful procedures, and is a better predictor of future reporting of pain than the initial pain report [12]. Therefore, it is key to improve our insights in helpful pain management strategies during painful procedures to counter the development and/or maintenance of negative memory biases. Interestingly, some pain management strategies may not be effective in reducing pain and anxiety experienced during needle procedures but do buffer against the development of negatively biased memories. For example, a study by Cohen et al. (2001) [19] examining the differential impact of distraction (i.e., nurse coaching and watching a movie), topical anesthetics (i.e., EMLA^®^ cream), and typical care (i.e., normal nurse–child interaction) during a three-injection vaccination series found that distraction or topical anesthetics did not result in less experienced pain or anxiety compared to typical care. However, the children recalled experiencing more pain and anxiety with typical care than with topical anesthetics or distraction.

Research has identified individual factors that influence children’s pain memory biases. For example, in a study by Marche et al. (2016) [20], findings showed that children with a poorer self-efficacy (i.e., their belief to cope effectively with experienced pain) towards the cold pressure task and other general pain experiences (e.g., headache) may experience less forgetting of negative aspects of a past painful experience compared to children with higher pain-related self-efficacy. Accordingly, it is possible that children who report lower levels of self-efficacy report less accurate pain memories compared to children who report high levels of self-efficacy. Further, children’s catastrophic thinking about pain influences the development of negative memory biases, with higher levels of child catastrophic worry about pain contributing to more negatively estimated pain memories post-surgery [21] as well as in contexts of acute experimental pain [22]. Additionally, parental variables have been shown to influence children’s pain memories; studies have demonstrated that parental catastrophic worry about their child’s pain is an even stronger predictor of children’s and parents’ pain memories following surgery than children’s catastrophic worry [11,21,23].

In order to buffer against the development of negatively biased pain memories and associated adverse outcomes over time, effective pain management strategies are imperative. Indeed, experienced pain is one of the most powerful predictors of negatively biased pain memories [11,24,25]. Various psychological interventions to mitigate procedure-related pain have been recommended, with distraction having a robust evidence base for pain reduction [1,5,26,27]. Distraction is characterized by shifting the child’s attention away from the stimulus (e.g., a needle) that may evoke an undesirable response (e.g., pain) towards a more pleasant stimulus (e.g., a game) that may evoke a positive response (e.g., laughter or enjoyment). As stated by Farrier et al. (2019) [2], distractions aimed at reducing pain should be stronger than the pain stimulus and include highly engaging components. Among other high-tech device distraction techniques (e.g., virtual reality), the use of a humanoid robot appears to be a promising distraction technique to reduce pain, fear, and distress during painful medical procedures [2,28,29,30]. One study by Lee-Krueger et al. [31] found no effect of a humanoid robot teaching deep breathing techniques prior to intravenous line (IV) placement, compared to standard care with Ametop©, on children’s experienced pain and fear during the IV line placement, but showed that children in the robot group were 5.04 times more likely to complete the IV induction. Humanoid robots are designed to include human characteristics, such as movements and appearance. They can be programmed to work with people and to communicate both verbally and nonverbally [28]. Children are particularly eager and receptive to engage with robots [28,32]. The potential of robot-led distraction in children with chronic diseases is multifold and includes its ability to achieve the following: (1) to be programmed in advance whereby the health care staff only needs to activate the robot, requiring minimal training; (2) to program the content of the robot’s speech and actions so that it is developmentally tailored to the age and abilities of the child; and (3) to make medical procedures less painful and distressing. However, the impact of distraction by means of a humanoid robot in children with chronic diseases undergoing needle procedures upon child pain-related memories remains to be investigated. Therefore, the current study aims were the following: (1) to evaluate the feasibility of implementing robot-led distraction in 8- to 12-year-old children with chronic diseases during needle procedures in a pediatric hospital outpatient setting; and (2) to explore preliminary effects of robot-led distraction on these children’s pain-related memory bias one week following the needle procedures (i.e., primary outcome), and on their experienced, recalled, and future expected pain intensity and pain-related fear, experienced and future expected self-efficacy, and experienced catastrophic worry during the needle procedure (i.e., secondary outcomes).

## 2. Materials and Methods

### 2.1. Study Design

This study was a randomized controlled pilot and feasibility trial. Clinical outcomes were assessed at baseline, immediately after the needle procedure, and one week later. This study was conducted in accordance with the ethical principles regarding human experimentation stated by the Declaration of Helsinki [33]. The study was approved by the Ethics Committee of the University Hospital Brussels on 20 February 2019 (B.U.N. 143201838477) and by the Ethics Committee of the University Hospital Ghent on November 3, 2020 (B.U.N. B143201838477). The protocol was registered at clinicaltrials.gov (ID: NCT04003701) in June 2019. The study is reported according to the CONSORT guidelines extension for randomized pilot and feasibility trials [34].

### 2.2. Protocol Deviations

Initially, this study was intended to be a randomized clinical trial (N = 104). However, this study was modified to be a pilot and feasibility trial (clinicaltrials.gov protocol registration formally updated on 24 May 2022) due to the following: (1) a prolonged shutdown of research activities in 2020 (March–June 2020) due to restrictions inherent to the COVID-19 pandemic; (2) limited funding for the project; and (3) the very low number of eligible children at the participating hospitals. Consequently, only a few initial pre-specified feasibility criteria were set in the official trial registration (e.g., needle procedure duration). Prior to any data analysis, consensus amongst the research team was reached to set additional feasibility outcomes, including study procedure variables (e.g., recruitment rate, dropout rate, and follow-up rate), needle-procedure-related characteristics (e.g., use of topical anesthetics), adverse events, intervention fidelity, intervention acceptability, and nurse perceptions of the intervention. All of these feasibility outcomes were clearly and consistently described and assessed during the study. The target sample size and statistical methods were adjusted to enable evaluation of intervention feasibility as well as preliminary efficacy. Accordingly, and in line with previous research with a similar approach [35], we aimed to recruit at least 10 participants per group to explore intervention feasibility and preliminarily assess the impact of robot-led distraction on pain-related memory bias. For the purposes of the current study, we focused exclusively on child-reported outcomes.

### 2.3. Participants

Participants included 22 children (8–12 years old) diagnosed with a chronic disease (e.g., chronic immune deficiency, juvenile idiopathic arthritis, and Crohn’s disease) that were scheduled to undergo a needle procedure at the pediatric outpatient clinics of the University Hospital Brussels and Ghent University Hospital as part of their treatment course. Children participated together with one of their parents (i.e., the parent who usually accompanied them during hospital visits). All procedures within the current study were needle procedures (e.g., medication admissions intravenously or through a residential portal catheter). No extra procedures were scheduled for the purpose of the current study. Stratified block randomization (stratified by sex (boy/girl), age (8–9 years/10–12 years), and recruitment site (University Hospital Brussels/Ghent University Hospital)) by an independent researcher (K.I.) was performed by means of the randomization tool ‘http://www.randomization.com’ (accessed on 2 September 2019) to assign patients to either the experimental group (i.e., robot-led distraction) or the control group (i.e., usual care). Group allocation was concealed in sequentially numbered sealed opaque envelopes, which the principal investigator (E.R.) opened just before delivering the intervention or control condition. Participants were not blinded, but because all questionnaires were self-reports, the investigators assume no bias exists depending on which group the participants were allocated to. Additionally, risk of bias assessment tools for randomized controlled trials, such as the RoB2 Cochrane tool [36], indicate that blinding is considered not appropriate in pragmatic trials in which the goal is to compare intervention strategies in individuals who are aware of their care. Clinical nurses were (unavoidably) aware of group allocation but were not involved in the outcome assessment.

Children were eligible to participate if they met the following criteria: (1) were diagnosed with a chronic disease; (2) were scheduled for a needle procedure as part of their treatment; (3) were aged 8–12 years old at the time of participation; (4) cohabited with the participating parent for the past 5 years or, in case of divided custody, for at least half of the time since divorce; and (5) were cognitively capable of completing the questionnaires. Children were excluded from participation if they had the following: (1) a neuro-developmental disorder (e.g., autism spectrum disorder); (2) a psychiatric disorder (e.g., anxiety disorder); or (3) a significant vision or hearing impairment (not remedied by, for example, glasses). In addition, children were excluded when (4) the child or parent was unable to fluently speak and read Dutch.

### 2.4. Procedure

A weekly e-mail was sent by the principal investigator (E.R.) to the research associate of both pediatric outpatient clinics (W.R. and E.M.) to obtain new registrations of eligible children. For each eligible child, the principal investigator introduced herself on the day of their hospital appointment and provided oral and written standardized study information, whereafter children and parents signed the informed consent form when giving assent/consent to participate. Study participation took place during their next treatment appointment; hence, the intervention never took place during the children’s first treatment appointment. 

Children and parents received baseline questionnaires at home via e-mail two days in advance, which they had to return no later than the night before study participation. In the baseline questionnaires, children reported on socio-demographic information (i.e., sex and date of birth), pain experiences (any) during the past 2 weeks, and catastrophic worry about pain by means of the Pain Catastrophizing Scale for Children (PCS-C) [37]. Parents reported on socio-demographic and medical information (to supplement diagnostic information obtained from the child’s medical record) about themselves and their child, and trait catastrophic worry about their child’s pain by means of the Pain Catastrophizing Scale for Parents (PCS-P) [38].

On the day of the procedure, one of the investigators welcomed the participants in a standard office room at the hospital 1 h before their appointment at the outpatient clinic. The child received another questionnaire with state (i.e., situation-specific) questions about the upcoming needle procedure, including self-reported anticipated pain intensity, pain-related fear, catastrophic thoughts about pain, and self-efficacy. Next, the principal investigator accompanied the child and parent into the procedure room and opened the sealed envelope revealing group allocation (out of participants’ sight). Children in the control group underwent the needle procedure with usual care, whereas children in the experimental group underwent the needle procedure with robot-led distraction. Immediately following the procedure, the children self-reported on their experienced levels of pain intensity, pain-related fear, catastrophic thoughts about pain, and self-efficacy. Children in the experimental group also reported on their experience with the robot. Finally, parents were provided with a sealed envelope that contained copies of the pain intensity and pain-related fear scales, which the child would need during the memory interview. Participants were informed that one of the investigators would call them one week later, without telling them what the phone call would be about, and were asked to ensure that the envelope remained sealed until this phone call. At the end of the study, after completion of the data collection, nurses were asked to report on their perception of the intervention.

### 2.5. Intervention

#### 2.5.1. Control Group: Usual Care 

In the control group, needle procedures were performed with usual care, including normal interactions between the child, parent, and nurse, as well as minimal distraction techniques that were usually performed in the outpatient clinics (e.g., counting, blowing, singing, watching television, listening to music, and playing a game on smartphone/tablet). More intensive distraction techniques (e.g., virtual reality, hospital clowns, or robot devices) were not allowed. The child was seated on the bed with both legs straight and arms stretched out along the body in a relaxed position. The nurse was situated at the puncture side and the parent was situated on the opposite side of the child next to the bed. The principal investigator sat down silently, approximately 2 m away from the bed in view of the child. At the end of the procedure, the nurse told the child that he/she did very well.

#### 2.5.2. Experimental Group: Robot-Led Distraction 

In the experimental group, a three-foot-tall humanoid robot NAO (H25 Academic Edition, Aldebaran Robotics, Paris, France) played a quiz game with the child throughout the needle procedure, starting from the moment that the nurse arrived at the bed with the medical instruments and ending when the entire needle procedure was completed. The child, nurse, and parent were positioned in the same way as in the control group. In addition, next to the child at the non-puncture side, the robot was sitting on a small mobile table at the child’s eye level, with the principal investigator standing behind it. The nurse was instructed to perform the needle procedure as usual, but the nurse and parent were only allowed to minimally interact with the child to optimize robot-led distraction. The robot was programmed to distract the child by playing a quiz game based on the child’s interests. The robot offered the child 5 possible quiz themes: Disney, superheroes, Ketnet (i.e., a Flemish television channel for children), sports, and geography. The quiz themes were developed based on the input of 20 healthy 8- to 12-year-old children and tested in terms of difficulty in 20 other healthy 8- to 12-year-old children. The purpose was that each quiz game was challenging enough, but that children would give a maximum of 1 wrong answer per quiz game on average, in order to activate their reward system as well. The child was asked by the robot to indicate his/her answer by pointing at the card of their choice, out of 3 cards presented by the principal investigator. On each card, a QR code was printed. The principal investigator showed the chosen QR code to the robot, after which the robot indicated whether the answer was correct or incorrect, using varied and positive language (e.g., “Good answer. You’re doing great. Here comes the next question.”). At the end of the procedure, the robot told the child he/she did very well.

### 2.6. Feasibility Outcomes

#### 2.6.1. Study Procedures

To evaluate the feasibility of the study procedures, we calculated the following outcomes: (1) enrollment rate, defined as the total number of participants that consented/assented to participate divided by the total number of patients that received oral and written information about the study; (2) dropout rate, defined as the total number of dropouts divided by the total number of participants that consented/assented to participate; (3) follow-up rate, defined as the total number of participants providing full study data divided by the total number of participants that received the intervention; (4) days between informed consent and participation; and (5) days between participation and completion of the memory interview.

#### 2.6.2. Needle-Procedure-Related Characteristics

For each child, the principal investigator recorded whether or not topical anesthetics were applied at the injection site, given that this is a highly efficacious pain management strategy [39], and whether or not mild sedation by means of Kalinox^®^ (i.e., an equimolar mixture of oxygen and nitrous oxide administered by mask induction) was used for the needle procedure. The use of topical anesthetics and/or sedation was not part of standard care at the participating hospitals, but rather determined on a case-by-case basis, and was allowed in both the experimental and control group. Further, the total duration of the needle procedures was measured using a chronometer, starting from the moment the nurse closed the door of the operating room and ending when the needle procedure was completed (e.g., intravenous drip fully attached around the arm or hand). The number of attempts to achieve a successful needle insertion was also recorded. Lastly, adverse events (e.g., failed needle insertion or blood loss) were recorded.

#### 2.6.3. Intervention Fidelity

Intervention fidelity (usual care vs. robot-led distraction) was evaluated according to the number of intervention components that were delivered as intended for each participant (usual care: 4 components, robot-led distraction: 5 components) (see Table 1). Each component was scored by the principal investigator as unsuccessful (coded as 0) or successful (coded as 1). Proportion scores were calculated as the sum of scores for each intervention divided by the total amount of components intended to be delivered for that intervention. We also calculated the proportion of participants for which the intervention was completely delivered as intended. Further, we described the amount of completed quiz games, chosen quiz themes, and score on completed quiz games for the experimental group. Lastly, we recorded distraction techniques used by nurses and parents in the control group, as well as possible additional strategies used by nurses and parents in the experimental group (e.g., counting and blowing), given that these are also pain management strategies known to reduce pain and distress [1,40].

#### 2.6.4. Intervention Acceptability

Immediately following the intervention, children in the experimental group reported on how much they liked playing the quiz game with the robot and how much they would like to have the robot with them again during a future needle procedure, using a numeric rating scale ranging from 0 (“not at all”) to 10 (“very much”). 

#### 2.6.5. Nurse Perception of the Intervention

After data collection was completed, each nurse who performed a needle procedure with the robot intervention received a brief questionnaire to report on their overall perceptions of the intervention. Nurses were asked about the following: (1) their beliefs regarding the aim of the intervention (open-ended question); (2) what went well during the intervention (open-ended question); (3) what did not go well/was challenging during the intervention (open-ended question); (4) how the children experienced the intervention (open-ended question); (5) to what extent they would be inclined to use the robot during future needle procedures (on a scale from 0 (“certainly not”) to 10 (“certainly yes”)); (6) how effective they believed the robot intervention was in terms of children’s pain reduction (on a scale from 0 (“ineffective”) to 10 (“highly effective”)); (7) how effective they believed the robot intervention was in terms of children’s pain-related fear reduction (on a scale from 0 (“ineffective”) to 10 (“highly effective”)); and (8) any other feedback on the intervention (open-ended question).

### 2.7. Clinical Outcomes

#### 2.7.1. Pain Intensity

Prior to (t_1_), immediately after (t_2_), and one week after (t_3_) the needle procedure, children’s anticipated (t_1_), experienced (t_2_), recalled (t_3_), and future expected (t_3_) pain intensity was assessed using the Faces Pain Scale-Revised (FPS-R) [41]. The FPS-R contains 6 age- and sex-neutral faces that illustrate increasing levels of pain intensity from the leftmost face (“no pain”: 0) to the rightmost face (“very much pain”: 10). Participants were instructed to indicate the face that corresponded most closely to their level of pain intensity. The FPS-R has demonstrated good psychometric properties [42] and has previously been used to assess initial and recalled pain in children aged 4 to 12 experiencing acute procedural pain [12,25,43].

#### 2.7.2. Pain-Related Fear

Prior to (t_1_), immediately after (t_2_), and one week after (t_3_) the needle procedure, children’s anticipated (t_1_), experienced (t_2_), recalled (t_3_), and future expected (t_3_) pain-related fear was assessed using the Children’s Fear Scale (CFS) [44]. The CFS contains 5 age- and sex-neutral faces that illustrate increasing levels of pain-related fear from the leftmost face (“no fear”: 0) to the rightmost face (“extremely fearful”: 4). Participants were instructed to indicate the face that corresponded best to their level of pain-related fear. The CFS has demonstrated good psychometric properties [44] and has previously been used to assess initial and recalled pain in children aged 4 to 12 experiencing acute procedural pain [12,25,43].

#### 2.7.3. Self-Efficacy

Prior to (t_1_), immediately after (t_2_), and one week after (t_3_) the needle procedure, children self-reported on anticipated (t_0_), experienced (t_1_), and future expected (t_2_) self-efficacy regarding the needle procedure. Children’s self-efficacy was assessed through one item about their perceived ability to cope with the upcoming/past needle procedure, using an 11-point numeric rating scale (NRS-11) ranging from 0 (“not at all”) to 10 (“extremely good”). Currently, there is no standard assessment of self-efficacy in children [45]; however, the NRS-11 has demonstrated sufficient measurement properties to measure pain-related outcomes in children from the age of 6 [42].

#### 2.7.4. State Pain Catastrophic Thoughts

Children—Prior to (t_1_) and immediately after (t_2_) the needle procedure, children self-reported on anticipated (t_1_) and experienced (t_2_) state (i.e., situation-specific) catastrophic thoughts about the needle procedure using an adaptation of the PCS-C [37]. In line with previous research [46,47,48,49,50], we used a state version of the PCS-C, including 3 items, with 1 adapted item from each of the 3 subscales (rumination: “To what extent do/did you keep thinking about how much pain the needle procedure could cause?”; magnification: “To what extent do/did you expect that, because of the pain, something serious would happen during the needle procedure?”; helplessness: “To what extent do/did you think you will not be able to endure the needle procedure because of the pain?”). Participants rated their anticipated or experienced catastrophic worry, respectively, regarding the needle procedure on an 11-point numeric rating scale ranging from 0 (“not at all”) to 10 (“extremely”). A mean score of these 3 items was calculated, ranging from 0 to 10. Good internal consistency and validity of the state PCS-C was demonstrated in community-based samples aged 8 to 18 years undergoing experimental pain [46]; however, research needs to examine the psychometric properties of this adapted version of the PCS-C in children experiencing acute procedural pain.

Parents—Prior to (t_1_) and immediately after (t_2_) the needle procedure, parents reported on anticipated (t_1_) and experienced (t_2_) state (i.e., situation-specific) catastrophic thoughts about their child’s needle procedure using an adaptation of the PCS-P [38]. Based on previous research [46,51,52,53], we used a state version of the PCS-P, including 3 items, with 1 adapted item from each of the 3 subscales (rumination: “To what extent do/did you keep thinking about how much pain the needle procedure could cause to your child?”; magnification: “To what extent do/did you expect that, because of the pain, something serious would happen to your child during the needle procedure?”; helplessness: “To what extent do/did you think your child will not be able to endure the needle procedure because of the pain?”). Parents rated their anticipated or experienced catastrophic thoughts, respectively, regarding their child’s needle procedure on an 11-point numeric rating scale ranging from 0 (“not at all”) to 10 (“extremely”). A mean score of these 3 items was calculated, ranging from 0 to 10. Good internal consistency and validity of the state PCS-P was demonstrated in community-based samples of parents of children aged 8 to 18 undergoing experimental pain [46] and parents of children with leukemia undergoing acute procedural pain [52,54].

#### 2.7.5. Memory Bias

Approximately one week after the procedure, participants were contacted by phone and a memory interview was conducted in accordance with previous research assessing pain-related memory in children [12,55,56,57]. In order to facilitate communication during the interview and to avoid introducing a confounding numeric rating scale, each FPS-R and CFS face was assigned a random letter of the alphabet below each face. Children were instructed to say aloud the letter that indicated the face of their choice. Memory bias was calculated as the difference between recalled pain intensity/pain-related fear and experienced pain intensity/pain-related fear reported immediately after the procedure by means of the FPS-R and CFS, respectively. A negative memory bias for pain intensity or pain-related fear was defined as recalling higher levels of pain intensity or pain-related fear during the memory interview as compared to the level of pain intensity or pain-related fear the child indicated immediately after the procedure (i.e., a positive value).

### 2.8. Statistical Analyses

Data were analyzed using IBM SPSS Statistics 28 (SPSS IBM, New York City, NY, USA). A *p*-value of less than 0.05 was considered statistically significant. Feasibility outcomes were analyzed in terms of frequencies and percentages (for dichotomous and categorical variables) or means and standard deviations (for continuous variables). Given the pilot and feasibility nature of this study, we conducted exploratory analyses on available clinical data, including independent t-tests to investigate between-group differences and visual presentations of distributions. Primary outcome measures were pain intensity memory bias and pain-related fear memory bias. Secondary outcome measures included experienced, recalled, and future expected pain intensity and pain-related fear, experienced and future expected self-efficacy, and experienced catastrophic worry about pain. We calculated Hedges’ g correction (with 0.2 = small effect size; 0.5 = medium effect size; 0.8 = large effect size), as this corrected effect size is preferred over Cohen’s d for small sample sizes [58,59], to estimate effect sizes for between-group differences and to be able to determine the sample size for a future definitive trial. Missing data were excluded from the analyses.

## 3. Results

### 3.1. Participant Characteristics

**Figure 1:**
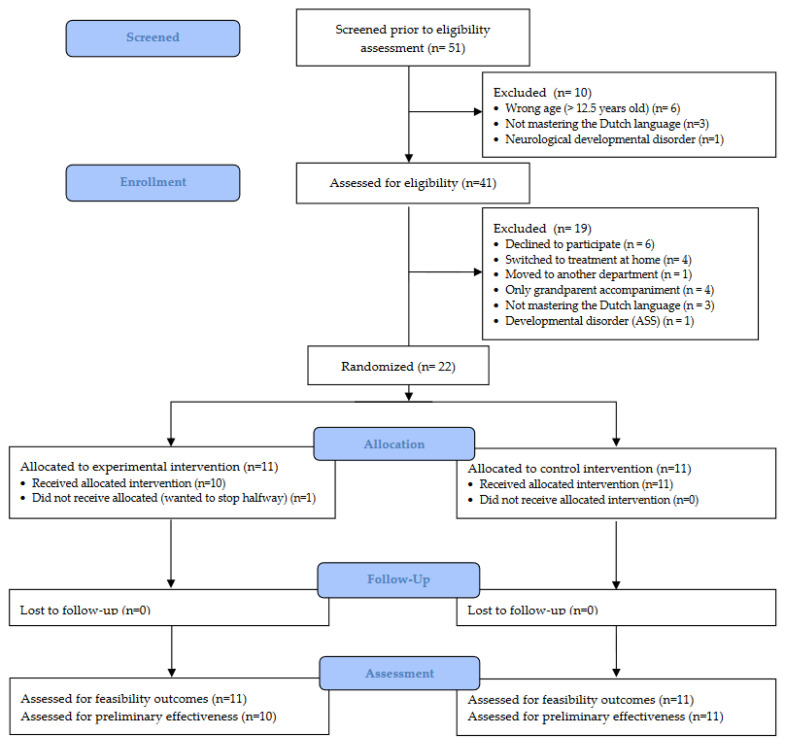
Participant flow chart.

A CONSORT extension Flow Diagram of patient recruitment and dropout is presented in Figure 1 [34]. Twenty-two children were randomized to either the experimental group or control group. Table 2 and Table 3 present socio-demographic and medical characteristics of the children and their parents, respectively. All children participated with one of their legal parents, and no children from the same family participated in the current study.

### 3.2. Feasibility Outcomes

#### 3.2.1. Study Procedures

Participants were recruited between September 2019–January 2022 and participated between October 2019–February 2022. Figure 1 presents the participant flow through the study. A total of 51 participants were screened for eligibility, resulting in a total number of 41 eligible children. A total of 39 children and their parents received oral and written information about the study; 1 child–parent dyad was not interested in receiving information about the study and 1 child was moved to another department. Of the 39 child–parent dyads that were informed about the study, 30 provided informed consent/assent, corresponding to an enrollment rate of 76.92%. Eight participants dropped out of the study, with switching to subcutaneous home treatment as the main reason (n = 4). In 1 participant, the intervention was not provided as intended, as the child wanted to stop interacting with the robot in the middle of the needle procedure. Subsequently, no post-needle-procedure data (t_1_ and t_2_) were collected for this child. Therefore, the dropout rate was 30%. All participants that received the intervention as intended completed the memory interview, resulting in a follow-up rate of 100%. Reasons for declining to participate and dropouts are presented in Figure 1. This resulted in a final sample of 22 children and one of their parents for the feasibility outcomes, and 21 children and one of their parents for the clinical outcomes. The mean number of days between informed consent and participation was 39.64 ± 45.85 days. The mean number of days between participation and the memory interview was 7.81 ± 1.78 days. 

#### 3.2.2. Needle-Procedure-Related Characteristics

In 13 out of 22 children (59.09%), with 6 children in the experimental group and 7 children in the control group, a topical anesthetic product was applied to the injection site prior to the needle procedure. Topical anesthetics included EMLA^®^ cream (n = 10), Rapydan^®^ (n = 2), or a cold spray (n = 1). No mild sedation by means of Kalinox^®^, or any other form of sedation, was used in any of the participants. The total duration of the medical procedures ranged from 2.42 to 19.00 min (7.42 ± 4.50): 8.76 ± 4.99 min for the experimental group and 6.08 ± 3.71 min for the control group. For the total sample, a mean number of 1.27 ± 0.55 attempts was needed to successfully insert the needle: 1.45 ± 0.69 in the experimental group and 1.09 ± 0.30 in the control group. No significant differences were found between the control group and experimental group in terms of total duration of the medical procedures (*p* = 0.169) or number of attempts for successful needle insertion (*p* = 0.124). One adverse event in the experimental group was recorded (i.e., complications with the needle procedure, see ‘Intervention Fidelity’).

#### 3.2.3. Intervention Fidelity

In total, 92.93% of intervention components were delivered as intended. In the control group, 95.45% of components were successfully delivered. For the experimental group, 90.91% of intervention components were successful for each participant. One child wanted to stop the robot-led distraction halfway through the needle procedure due to complications with the needle insertion (i.e., insertion into an artery instead of a vein, resulting in blood loss) and resulting increased distress. This resulted in a total of 90.91% of children in the experimental group for which the intervention was delivered as intended. There was only one unsuccessful component in the control group (n = 2), being the standardized position of the child, parent, nurse(s), and investigator in the procedure room (e.g., mother wanted to stand against the wall further from the child’s bed). In the experimental group, unsuccessful components included the following: standardized positioning of child, parent, nurse(s), investigator, and robot in the procedure room (n = 1); correct timing of the initiation of the robot intervention (n = 1); robot-led distraction throughout the full duration of the needle procedure (n = 1); and minimal interaction between the nurse, child, and parent (n = 2). 

Of the 11 children who participated in the experimental group, 6 (54.55%) children completed one quiz game, 3 (27.27%) children completed two quiz games, 1 (9.09%) child completed three quiz games, and 1 (9.09%) child did not complete any quiz game (i.e., the child that wanted to stop the intervention). The most frequently chosen quiz theme was sports, chosen by 6 (54.55%) out of 11 children for their first or second game, followed by Disney (4 (36.36%) children), superheroes (3 (27.27%) children), geography (2 (18.18%) children), and Ketnet (i.e., a Flemish television channel for children) (1 (9.09%) child). Of the 15 quiz games that were completed in total, children had the maximum score (i.e., all answers correct) in 12 (80%) games, and in the other games only one question was answered incorrectly. In 10 of the 22 participants, who were all assigned to the control group, some distraction strategies were used. No additional distraction strategies beyond the humanoid robot were observed in the experimental group. Specifically, in the control group, the nurse counted from three to one, followed by blowing by the child, in four (36.36%) children. In six (54.55%) other children, the nurse counted from three to one without subsequent blowing. In five (45.45%) children, the parent used non-pain-attending verbalizations (i.e., verbalizations toward the child that are not focused on the child’s pain, e.g., talking about fun plans for the weekend). Two (18.18%) parents held their child’s hand during the procedure, one (9.09%) consciously looked away from the needle procedure, and one (9.09%) watched television during the needle procedure.

#### 3.2.4. Intervention Acceptability

Overall, the robot-led distraction intervention was judged to be highly acceptable. Children in the experimental group reported a mean score of 9.45 ± 0.69 out of 10 for the question “How much did you like playing the quiz game with the robot?”, and a mean score of 8.73 ± 2.20 out of 10 for the question “How much would you like to have the robot with you again during a future needle procedure?”.

#### 3.2.5. Nurse Perception of the Intervention

A total of five nurses performed needle procedures with robot-led distraction and reported on their perceptions of the intervention. One nurse transferred to another employer during the study and could not be reached for completion of the questionnaire, resulting in data obtained from four nurses. Overall, nurses understood that the purpose of the intervention was to distract the children throughout the needle procedure and found that children, for those who were open to it, responded well and enthusiastically to the robot. However, multiple challenges and disadvantages of the intervention were reported by the nurses, including the following: (1) finding it difficult to not talk to or comfort the child as they are used to; (2) the robot did not always quickly register the children’s answers; (3) the robot was not always easy to understand, which interfered with the robot having smooth interactions with the child; (4) the introduction of the robot to the child took too much time; (5) the intervention is a good method, but is not applicable for each child; (6) there is a need for one additional person besides the nurse to control the robot during the procedure; (7) the robot was unnecessary/did not add value; (8) the robot did not have a high ‘cuddle factor’; (9) belief that topical anesthetics contribute more to pain reduction than the robot; (10) belief that the needle procedure took more time compared to procedures with other forms of distraction; and (11) the intervention was not perceived as running smoothly and fluidly due to the tight protocol. On a scale from 0 to 10, nurses’ mean scores for how much they were inclined to use the robot during future needle procedures, perceived effectiveness of the robot intervention in terms of pain reduction, and perceived effectiveness of the robot intervention in terms of pain reduction were 6.00 ± 2.55, 6.75 ± 1.64, and 5.50 ± 2.06, respectively. Of note, the most feedback and also the most negative feedback was reported by one of the nurses. A table summarizing the nurse perceptions of the intervention can be found as Supplementary Material (Table S1). 

### 3.3. Clinical Outcomes

No between-group differences were observed for any of the clinical outcomes at baseline or just before the needle procedure, including children’s trait pain catastrophizing (PCS-C trait; *p* = 0.85), state pain catastrophizing (PCS-C state; *p* = 0.35), anticipated pain intensity (FPS-R; *p* = 0.67), anticipated pain-related fear (CFS; *p* = 0.87), anticipated self-efficacy (NRS-11; *p* = 0.32), and parents’ trait pain catastrophizing (PCS-P trait; *p* = 0.98) and state pain catastrophizing (PCS-P state, *p* = 0.54). A medium effect size on memory bias for pain intensity (Hedges’ g = 0.70) and a very small effect size on memory bias for pain-related fear (Hedges’ g = 0.09) were found, in favor of the robot-led distraction intervention. In addition, for experienced self-efficacy, a medium effect size (Hedges’ g = −0.46) was shown in favor of robot-led distraction. For the other secondary outcome measures, small to medium effect sizes were found in favor of usual care. Results are presented in Table 4.

Table 5 presents an overview of the number of children per group and in the total sample that can be categorized as having a negative memory bias (i.e., t_3_ − t_2_ = positive value), a positive memory bias (i.e., t_3_ − t_2_ = negative value), or an accurate memory (i.e., t_3_ − t_2_ = 0) of the needle procedure. In line with the effect sizes, for memory bias for pain intensity a trend is visible in favor of the experimental group (i.e., less children with a negative memory bias and more children with an accurate memory or positive memory bias).

## 4. Discussion

This pilot study examined the feasibility of a robot-led distraction intervention vs. usual care to reduce the negative impact of needle procedures in children with chronic diseases. Overall, the data collection methods as well as the robot-led distraction intervention were deemed to be feasible and perceived to be highly acceptable by the children. Nurses’ perceptions of the intervention were mixed, indicating several challenges that provide suggestions for protocol adaptations for a future trial. Preliminary statistical analyses indicated a medium effect size on memory bias for pain intensity and a very small effect size on memory bias for pain-related fear in favor of the robot-led distraction intervention.

### 4.1. Intervention Feasibility

The enrollment rate (76.92%) was good, and the follow-up rate (100%) was excellent. The dropout rate (30%) was relatively high, but caution in interpreting these percentages is warranted because of the small sample size. The treatment course of children with chronic diseases can be unpredictable, and intervals between treatment times varied from 3 to 6 weeks. As a result, some children switched from intravenous hospital treatment to subcutaneous home treatment between informed consent and study participation. In other children, other unforeseen circumstances (e.g., mother switched jobs and only the grandparent could accompany the child during future hospital visits) caused them to drop out of the study after providing informed consent. Therefore, we recommend to account for a higher dropout rate (e.g., 30%) in future trials.

Overall, delivery of intervention components was successful, and children’s perceptions of the acceptability of the intervention was high. The level of difficulty of the questions posed by the robot was low to moderate, so that many children could answer all the questions correctly while still requiring some time to think about the correct answer, indicating that the questions provided some level of cognitive challenge. Most children completed one quiz game throughout the needle procedure, which was anticipated, as the length of one complete quiz game was based on the mean length of four needle procedures of non-participants that were observed during the preparation phase of the current study. 

Although nurses provided positive feedback about the intervention, they also reported challenges and suggestions for protocol adjustments that can be used to guide future work. For example, some nurses indicated that it was difficult to not talk to or comfort the child during the robot-led distraction intervention as they were previously used to; that they thought that the intervention was not easy to implement in clinical practice because of the need for an additional person to control the robot; and that the intervention might be of little added value to other existing distraction/pain management strategies (e.g., topical anesthetics). Further, one nurse reported that completing the needle procedure in the presence of the robot took longer as compared to usual care; however, this was not confirmed by statistical analyses (i.e., no significant between-group difference in total procedure duration). Nurse perceptions in the current study are in line with other research on psychological pain management strategies during needle procedures. For example, a study by Braithwaite et al. (2022) [35] investigating the feasibility of a divided attention and positive memory reframing intervention compared to usual care to reduce needle pain and distress in children revealed similar concerns of nurses, such as that needle procedures combined with a pain management intervention (e.g., robot-led distraction in the current study) take more time as compared to other strategies they usually apply (e.g., blowing), and that it is hard to change their behavior towards the child during the needle procedure (e.g., not comforting the child during the intervention as usual) in adherence to the study protocol. Overall, the feedback and challenges raised by the nurses call for input of nurses in the methodology of future work. Intervention co-creation offers a promising way to improve upon the feasibility of the intervention as well as the implementation in clinical practice (see ‘Recommendations and Future Directions’).

### 4.2. Clinical Effectiveness

In sum, preliminary results on the clinical effectiveness of robot-led distraction supported the promising potential of robot-led distraction upon pain-related memories in the context of needle procedures in children with chronic diseases and supported continued inquiry towards improved pain management protocols for children during needle procedures. Specifically, we observed a medium effect size and visual trend for pain intensity memory bias in favor of the experimental group (i.e., less children with a negative memory bias and more children with an accurate memory or positive memory bias). For pain-related fear memory bias, only a very small effect size could be observed, although also in favor of the robot-led distraction intervention. These differential findings for sensory (i.e., pain intensity) and affective (i.e., pain-related fear) aspects of children’s pain-related memories underline that, although both aspects of a child’s pain experience are associated with each other, these two dimensions and their relations with other child pain outcomes may diverge [60]. Research on children’s memory for pain reveals key differences between recall of pain intensity as compared to pain affect, providing support for the distinctiveness and relative clinical significance of children’s recall of sensory vs. affective dimensions of pain. In fact, weak correlations between both dimensions of recalled pain are frequently observed (see [61]). Findings of the current study underscore the importance of a multidimensional (memory for) pain assessment (i.e., including measures for both sensory and affective aspects of pain; also see [12,16,56,57,60,62,63]). This being said, findings of the current study tentatively suggest the promising potential of robot-led distraction to improve pain-related memory bias development in children with chronic diseases in the context of needle procedures. These preliminary findings on the effectiveness of robot-led distraction on children’s pain-related memory bias are important, as children’s negatively biased memories of painful experiences are considered to have important implications for children’s coping during subsequent painful procedures, are a better predictor of future pain reporting than the initial pain report [12], and are suggested to be involved in the development or maintenance of pain problems later in life [11,12,13].

The observed effect sizes for pain intensity memory bias and fear memory bias confirm and extend previous findings indicating the beneficial impact of robot-led distraction interventions on children’s pain [2,28], fear [2], distress [28,30], and negative emotions [64] during medical procedures. However, a number of tentative explanations may account for the non-significance of the observed effects in our study. First, the sample size (N = 21) was small, reducing the power of the study and increasing the margin of error. Second, overall low levels of pain intensity and pain-related fear were reported in both the experimental and control group, potentially inducing a floor effect and limiting the robot’s ability to improve pain-related outcomes. Next, it is important to consider the study population; given the need to repeatedly undergo needle procedures, children with chronic diseases and parents may have already searched for, utilized, and developed preferences for particular strategies to cope with unpleasant medical procedures. Thus, although the acceptability data suggested the robot-led distraction intervention to be highly acceptable, children in the experimental group were required to be open to a new strategy (i.e., robot-led distraction), which may have been a challenging adjustment for some children. Moreover, group assignment was not revealed until the start of the needle procedure, and children were not aware of what the robot-intervention would entail, creating an extra surprising effect, which may have induced additional stress for some. Alternatively, it is equally possible that children in the control group felt disadvantaged or disappointed because they did not get to see the robot. Importantly, except for the discontinuation of the intervention in one child due to complications during the needle procedure, no adverse effects on any of the clinical outcome measures were found in the current study. Another explanation might lie in the distraction strategy itself (i.e., a quiz game with the robot). The robot-led distraction intervention was created such that the distraction would be highly engaging and active [65], in order to draw them towards the distraction more strongly than towards the needle procedure. However, the strength of certain distraction strategies might be different for each individual child and depend on other contextual factors (e.g., parental responses), hence it is possible that for some children the robot intervention was not sufficiently engaging or attractive to stay focused on the robot and distracted away from the painful stimulus. For example, for some children, the robot dancing or singing instead of playing a cognitive game would be more effective [29]. Additionally, focused attention is rarely static and is dependent on context. Indeed, the more intense the painful stimulus is perceived to be, the less effective the distraction will be [66]. This was illustrated by the child who wanted to stop interacting with the robot in the middle of the needle procedure, because of procedural complications that led the child to perceive the needle as being more painful and distressing. Lastly, allowing other low- and high-technology distraction strategies in the control group, such as watching television, might have decreased the likelihood of finding differential effects of robot-led distraction vs. usual care. Indeed, a recent systematic review [40] categorized watching television or playing games on a smartphone under the same distraction category (i.e., “high-technology distraction”) as the use of a humanoid robot, and stated that low- vs. high-technology distractors do not have a differential impact upon needle-related pain and distress in children. 

Previous research has also found null effects of robot-led distraction for children’s needle procedures. For example, Lee-Krueger et al. (2021) [31] examined the impact of a humanoid robot programmed to teach deep breathing in addition to standard care (i.e., including topical anesthetic cream) vs. standard care alone on children’s pain and fear during intravenous line placement. Similarly, Ali et al. (2021) [30] examined the impact of humanoid robot-led distraction in addition to standard care (i.e., including topical anesthetic cream) vs. standard care on pain and distress in children undergoing intravenous insertion. In these studies, no impact of robot-led interventions was found upon experienced pain [30,31] and fear [31]. Importantly, both studies did not investigate the impact of robot-led interventions upon children’s pain-related memories. Taken together with the current findings, several areas for future research are identified. The use of a humanoid robot shows potential as a feasible distraction intervention for children with chronic diseases during needle procedures. However, to achieve optimal success, the intervention should be tailored to the child’s individual needs which could be achieved to through co-creation with children, parents, nurses, and child-life specialists (see ‘Recommendations and Future Directions’).

### 4.3. Recommendations and Future Directions

Before proceeding to a full RCT, future qualitative and quantitative research should examine the following: (1) which types of activities/behaviors led by the robot (e.g., game, dancing, singing, or storytelling) are preferred by children; (2) which type of robot-led distraction is most effective in capturing and maintaining children’s attention under certain conditions (e.g., complications during needle procedures) given that the valence of the distraction needs to be stronger than the painful stimulus; (3) what is the most optimal way of engaging the child in the intervention in order to optimize clinical outcomes; and (4) what are nurses’ and parents’ perspectives on how to optimize the robot intervention. For example, studies investigating the effects of robot-led distraction interventions [2,29,31], including the present study, let an investigator, research associate, or doctor control the robot. However, parents may like to control the robot themselves to empower them in supporting their child to cope with the procedure, which matches the nurses’ feedback about the need for an extra person to control the robot. Alternatively, although this was not the case in any of the participants in the current study, children are sometimes supported by a child-life specialist to help them in coping with painful medical procedures. In case a child is used to be supported by such child-life specialist, this person could control the robot as well. Therefore, a first step in future work could be the co-creation of the robot-led distraction intervention, with children, parents, nurses, and child-life specialists as key stakeholders and co-developers of the intervention. Co-creation, co-production, or co-design of research interventions can be broadly defined as the process of involving stakeholders in the development of interventions, defining directions and purposes, and solving problems together [67,68,69]. Through this approach, the ideas, needs, and interests of all relevant stakeholders (i.e., child, parents, nurses, child-life specialists, and research team) can be merged into one or more co-created robot-led distraction intervention that participants can choose during needle procedures so that they are tailored to their individual needs. Additionally, findings suggest that nurses may benefit from extra experience in performing medical procedures with the robot intervention in order to increase acceptability and successful implementation. Further, as the population of the current study concerned children with various chronic diseases, with variable time since diagnoses and treatment initiation, future studies should assess and control for previous experiences with needle procedures and other medical procedures within the statistical analyses. Last, given the small sample size and the nearly equal number of children using topical anesthetics in the experimental (*n* = 6) and control group (*n* = 7), no subgroup analyses were performed to see if there was a differential effect of the robot-led intervention in children who did vs. did not use topical anesthetics. Therefore, we recommend that future studies use topical anesthetics for all children to be able to examine the added benefit of robot-led distraction.

### 4.4. Strengths and Limitations

Strengths of this pilot and feasibility study include protocol pre-registration and rigorous reporting with adherence to the CONSORT guidelines extension for randomized pilot and feasibility trials [34]. Not all feasibility criteria were pre-registered, but all were determined by the research team throughout the study prior to data analysis. Another strength of this study is that the control group received usual care, enabling us to analyze the effectiveness of the robot-led distraction intervention compared to usual care pain management strategies (e.g., blowing, counting, and non-high-tech distraction). Further, the robot-led distraction intervention can easily be implemented in clinical practice without any need for an additional person, in contrast to what was indicated by two of the nurses as a limitation (“need for one additional person besides the nurse to control the robot”). With a minimal introduction at first use, parents could replace the investigator in controlling the robot during the procedure, which could serve to empower them and their child, and improve child coping. This is an important strength, as Belgian hospitals have been struggling with a large staff shortage and high workload among nurses, while the use of a humanoid robot to distract children during needling procedures could reduce the burden on health care staff. Lastly, feasibility outcomes reported in the current study provide clear directions for protocol amendments and further pilot testing.

There were also limitations that inform avenues for future research. First, only five nurses performed the needle procedures, hence variability and representativeness of nurse perceptions were limited, and other feasibility outcomes related to differing skill levels and experience of nurses may not have been captured. Additionally, one of the four nurses provided substantially more feedback on her perceptions of the robot-led distraction as compared to the other nurses. The majority of this nurse’s feedback was negative, while the feedback of the other three nurses was overall positive, partly biasing the overall impression of the nurse’s perceptions of the intervention. Second, recruitment for the current study was complicated due to several reasons, resulting in a small sample size after a relatively long recruitment period. The main reasons were the following: (1) a prolonged shutdown of research activities in 2020 (March–June 2020) due to restrictions inherent to the COVID-19 pandemic; (2) limited funding for the project; and (3) the very low number of eligible children at the participating hospitals. Third, technical aspects of the robot intervention should be optimized. Specifically, when the robot had to wait too long to start introducing the game or the lighting was not optimal, the robot sometimes did not smoothly scan the QR codes. While major technical problems or malfunctions did not occur, this technical aspect could be improved. Fourth, unlike many other pain management strategies that are costless (e.g., singing and distracting with humor) or cost little (e.g., blowing bubbles, books, and music), the cost of a humanoid robot is high. Other non-financial costs of robot-led distraction, such as training to program and control the robot and time investment during needle procedures, are considered limited relative to the potential benefits of robot-led distraction. Fifth, in the control group, other minimal distraction strategies were allowed (e.g., singing, listening to music, playing a game on their smartphone, and watching television). Given that the literature [40,70] indicates beneficial yet no differential effects of low- vs. high-technology distraction strategies upon experienced pain and distress in children undergoing needle procedures, allowing certain distraction strategies in the control group might have reduced the likelihood of finding stronger effects of robot-led distraction as compared to usual care upon our clinical outcome measures. However, the present study wanted to compare the effect of robot-led distraction vs. usual care, including usual care pain management strategies of any kind, and not the effect of usual care + robot-led distraction vs. usual care. Sixth, patients were not blinded from group allocation. In future research, it may be recommended to describe the study as an investigation of the effects of different possible distraction strategies, without explicitly stating robot-led distraction as a possible strategy, and to debrief patients about the actual research question at the end of the study. Additionally, success of participant blinding could be examined at the end of the study by asking participants whether they think they received the experimental or control intervention, including a percentage of certainty (i.e., 50% certainty indicating a pure guess) [71]. Further limitations include those inherent to many feasibility studies, namely that it was not powered for efficacy analyses. 

## 5. Conclusions

Protocol adjustments and further pilot and feasibility testing are recommended before proceeding to a full RCT. Recommended adjustments may include using a co-creation approach to develop the most optimal robot-led distraction intervention(s) based on feedback of children, parents, nurses, and child-life specialists. Further adjustments could include more extensive information and training of nurses in performing needle procedures in the presence of the robot before progressing to actual study participation, speed-training parents or child-life specialists in controlling the robot instead of the investigator, and improving technical aspects of the robot intervention (e.g., optimization of response registration). Given that robot-led distraction can be easily implemented in various clinical settings and pediatric populations, this suggests that this intervention holds promise for improving needle procedures for children with chronic diseases in need of life-long treatment.

## Figures and Tables

**Table 1:** Components of the control and experimental intervention.

Control Intervention (i.e., Usual Care)	Experimental Intervention (i.e., Robot-Led Distraction)
1. Standardized positioning of child, parent, nurse(s), and investigator in the procedure room 2. Exclusively non-intensive distraction (e.g., counting, blowing, singing, …) 3. Normal interaction between nurse, child, and parent 4. Nurse ending with “You did very well!”	1. Standardized positioning of child, parent, nurse(s), investigator, and robot in the procedure room 2. Correct timing of initiating robot intervention (i.e., nurse closed door after entering procedure room) 3. Robot-led distraction throughout full duration of needle procedure (before, during, and after needle insertion) 4. Minimal interaction between nurse, child, and parent 5. Robot ending with “You did very well!”

**Table 2:** Demographic and medical characteristics of children.

Characteristic		EG (n = 11)	CG (n = 11)	Total (N = 22)
Child age; M (SD)		9.36 (1.36)	9.45 (1.37)	9.41 (1.33)
Child sex; n (%)	Boys Girls	6 (54.55) 5 (45.45)	4 (36.36) 7 (63.64)	10 (45.45) 12 (54.54)
Diagnosis; n (%)	Chronic immune deficiency Auto-immune/-inflammatory disease Metabolic disease Inflammatory bowel disease Combination	6 (54.55) 3 (27.27) 0 (0.00) 1 (9.09) 0 (0.00)	6 (54.55) 2 (18.18) 3 (27.27) 0 (0.00) 1 (9.09)	12 (54.55) 5 (22.73) 3 (13.64) 1 (13.64) 1 (13.64)
Time since diagnosis (months); M (SD)		58.45 (30.86)	54.55 (29.35)	56.50 (29.46)
Pain in the last 2 weeks child; n (%)	Yes No	7 (63.64) 4 (36.36)	7 (63.64) 4 (36.36)	14 (63.64) 8 (36.36)
Pain intensity last 2 weeks child; n (%)	No pain Little pain Moderate pain Much pain Very much pain	4 (36.36) 0 (0.00) 6 (54.55) 1 (9.09) 0 (0.00)	4 (36.36) 1 (9.09) 6 (54.55) 0 (0.00) 0 (0.00)	8 (36.36) 1 (4.55) 12 (54.54) 1 (4.55) 0 (0.00)
Pain frequency last 2 weeks child; n (%)	Never Once Few times Often Continuously	4 (36.36) 1 (9.09) 4 (36.36) 2 (18.18) 0 (0.00)	4 (36.36) 0 (0.00) 7 (63.64) 0 (0.00) 0 (0.00)	8 (36.36) 1 (4.55) 11 (50.00) 2 (9.09) 0 (0.00)
Chronic pain child (> 3 months); n (%)	Yes No	2 (18.18) 9 (81.82)	4 (36.36) 7 (63.64)	6 (27.27) 16 (72.73)

EG = experimental group; CG = control group; M = mean; SD = standard deviation; chronic immune deficiency (e.g., hypogammaglobulinemia); auto-immune/-inflammatory disease (e.g., juvenile idiopathic arthritis); metabolic disease (e.g., Pompe disease); inflammatory bowel disease (e.g., colitis ulcerosa).

**Table 3:** Demographic and medical characteristics of parents.

Characteristic		EG (n = 11)	CG (n = 11)	Total (N = 22)
Parent age; M (SD)		39.82 (4.26)	40.27 (5.27)	40.05 (4.69)
Parent sex; n (%)	Fathers Mothers	2 (18.18) 9 (81.82)	1 (9.09) 10 (90.91)	3 (13.64) 19 (86.36)
Health status parent; n (%)	Excellent Very good Good Moderate Poor	1 (9.09) 5 (45.45) 5 (45.45) 0 (0.00) 0 (0.00)	1 (9.09) 4 (36.36) 5 (45.45) 1 (9.09) 0 (0.00)	2 (9.09) 9 (40.91) 10 (45.45) 1 (4.55) 0 (0.00)
Family status; n (%)	Married or cohabiting Divorced Widow(er) Single parent or unmarried Newly assembled family	7 (63.64) 3 (27.27) 0 (0.00) 1 (9.09) 0 (0.00)	11 (100.00) 0 (0.00) 0 (0.00) 0 (0.00) 0 (0.00)	18 (81.82) 3 (13.64) 0 (0.00) 1 (4.55) 0 (0.00)
Education level parent; n (%)	Primary education (≤ 12yo) Lower secondary education (≤ 14yo) Higher secondary education (≤ 18yo) Higher education (bachelor/master)	2 (18.18) 0 (0.00) 4 (36.36) 5 (45.45)	1 (9.09) 0 (0.00) 4 (36.36) 6 (54.55)	3 (13.64) 0 (0.00) 8 (36.36) 11 (50.00)
Occupation parent; n (%)	Housewife/househusband Laborer Employee Liberal profession Self-employed Manager position Unemployed	0 (0.00) 1 (9.09) 6 (54.55) 0 (0.00) 2 (18.18) 1 (9.09%) 1 (9.09%)	0 (0.00) 1 (9.09) 4 (36.36) 0 (0.00) 3 (27.27) 3 (27.27%) 0 (0.00%)	0 (0.00) 2 (9.09) 10 (45.45) 0 (0.00) 5 (22.73) 4 (18.18) 1 (4.55)

EG = experimental group; CG = control group; M = mean; SD = standard deviation; yo = years old.

**Table 4:** Preliminary results for child clinical outcomes.

Outcome	Total Sample (N = 21) M (SD)	EG (n = 10) M (SD)	CG (n = 11) M (SD)	*p*-Value	95% CI (Lower Upper)	Hedges’ g
Experienced pain intensity (t_2_)	2.10 (2.64)	3.00 (3.43)	1.27 (1.35)	0.163	(−4.27, 0.81)	−0.50
Experienced pain-related fear (t_2_)	0.71 (1.01)	1.10 (1.29)	0.36 (0.51)	0.117	(−1.69, 0.215)	−0.74
Experienced self-efficacy (t_2_)	7.57 (2.94)	8.30 (1.77)	6.91 (3.67)	0.280	(−4.04, 1.26)	−0.46
Experienced pain catastrophizing (t_2_)	2.16 (2.47)	2.80 (3.27)	1.58 (1.36)	0.293	(−3.65, 1.20)	−0.48
Recalled pain intensity (t_3_)	2.67 (2.13)	3.00 (2.71)	2.36 (1.50)	0.522	(−2.72, 1.45)	−0.28
Recalled pain-related fear (t_3_)	1.05 (1.12)	1.40 (1.35)	0.73 (0.79)	0.174	(−1.67, 0.32)	−0.59
Future pain intensity (t_3_)	2.95 (2.42)	3.20 (2.70)	2.73 (2.24)	0.666	(−2.73, 1.79)	−0.18
Future pain-related fear (t_3_)	1.05 (1.16)	1.30 (1.50)	0.82 (0.75)	0.375	(−1.61, 0.65)	−0.40
Future self-efficacy (t_3_)	8.24 (1.84)	7.80 (2.20)	8.64 (1.43)	0.311	(−0.84, 2.52)	0.44
MB pain intensity (t_3_ − t_2_)	0.57 (1.57)	0.00 (1.33)	1.09 (1.64)	0.113	(−0.28, 2.47)	0.70
MB pain-related fear (t_3_ − t_2_)	0.33 (.66)	0.30 (.82)	0.36 (.50)	0.831	(−0.55, 0.71)	0.09

t_1_ = before needle procedure; t_2_ = immediately after needle procedure; t_3_ = memory interview; EG = experimental group; CG = control group; M = mean; SD = standard deviation; Hedges’ g = Hedges’ g corrected effect size for small sample sizes.

**Table 5:** Overview of memory of number of participants per category of memory biases.

Outcome		Total Sample (N = 21) n (%)	EG (n = 10) n (%)	CG (n = 11) n (%)
MB pain intensity	Negative (+ value)	8 (38.10)	2 (20.00)	6 (54.55)
	Accurate (zero)	10 (47.62)	6 (60.00)	4 (36.36)
	Positive (− value)	3 (14.29)	2 (20.00)	1 (9.09)
MB pain-related fear	Negative (+ value)	7 (33.33)	3 (30.00)	4 (36.36)
	Accurate (zero)	13 (61.90)	6 (60.00)	7 (63.64)
	Positive (− value)	1 (4.76)	1 (10.00)	0 (0.00)

EG = experimental group; CG = control group.

## Data Availability

The data presented in this study are available on request from the corresponding author.

## Supplementary Materials

The following supporting information can be downloaded at: https://www.mdpi.com/article/10.3390/children9111762/s1, Table S1: Summary of nurse perceptions of interventions.
