# Supplementary material for: The Effect of Robot-Led Distraction during Needle Procedures on Pain-Related Memory Bias in Children with Chronic Diseases: A Pilot and Feasibility Study
Source: Children (Basel). 2022 Nov 17;9(11):1762. doi: 10.3390/children9111762 (PMC9688830; doi:10.3390/children9111762)
Supplement: Supplementary file 1 [file children-09-01762-s001.zip › children-1999892-supplementary.pdf]

SUPPLEMENTARY MATERIAL

TableS1. Summary of nurse perceptions of interventions.

Item	Nurse response (n=4)
What was, according to you, the purpose of the robot intervention?	Distraction of the child (1-4)<br>Reducing pain and anxiety during a needle procedure (2)
When you think of the needle procedure(s) you performed with the robot intervention, what went well?	Procedure went smoot (1,3)<br>I could focus on the procedure technique (1)<br>Depending on the individual child, the robot managed to distract the infant, causing them to experience less pain (2)<br>Children were distracted (4)
When you think of the needle procedure(s) you performed with the robot intervention, what did not go well or what was challenging?	Not talking to the child or counting 3 2 1 during the procedure was an adjustment (1,2)<br>When the child did not immediately succeed in paying attention to the robot, I wanted to help him to return attention to the robot, but it was difficult to know whether we were allowed to do that or not (2)<br>Your instinct is to start comforting the child but that was not possible (2)<br>The robot did not always fluently register the children's answers to the quiz questions so that the child's attention went away from the robot (1)<br>The robot was not always easy to understand which made it difficult for the children to interact with it smoothly (2)<br>The introduction of the robot was a bit too long (4)
How did the children overall perceived the robot intervention according to you?	Good, positive (1)<br>Some children are more easily impressed by a robot than others. The robot distracted some children well, others were less susceptible to it (2)<br>Enthusiastic (3)<br>Cool and fun, for children who were open to it (4)
Do you have any other feedback on the robot intervention?	Good method, but not applicable for each child (1)<br>Need for one additional person besides the nurse to control the robot (1,2)<br>I personally find the whole procedure unnecessary and do not see the added value (2)<br>Nurses can anticipate anxiety much better than a machine (2)<br>Robot had no high 'cuddle factor', perhaps a soft/stuffed version would be more successful? (2)<br>I believe that the use of a local anesthetic contributes more to a good distraction and pain reduction than the robot (2)<br>I felt that the entire procedure took more time than other forms of distraction already used (2)<br>The robot is a fun attraction which provides a good distraction (3)<br>Because of the tight protocol, things were perhaps a bit awkward/stiff (3)
Would you be inclined to use the robot during future needle procedures? (NRS-11); M(SD)	6.00 (2.55)
According to you, how effective is the robot intervention in terms of pain reduction for children undergoing a needle procedure? (NRS-11); M(SD)	6.75 (1.64)
According to you, how effective is the robot intervention in terms of pain-related fear reduction for children undergoing a needle procedure? (NRS-11); M(SD)	5.50 (2.06)

1 = nurse 1; 2 = nurse 2; 3= nurse 3; 4 = nurse 4. Results presented in this table summarize the statements reported by the nurses after data collection was completed. Of note; most and also the most negative feedback was reported by nurse 2.
